# Increasing Engagement in the Electronic Framingham Heart Study: Factorial Randomized Controlled Trial

**DOI:** 10.2196/40784

**Published:** 2023-01-20

**Authors:** Ludovic Trinquart, Chunyu Liu, David D McManus, Christopher Nowak, Honghuang Lin, Nicole L Spartano, Belinda Borrelli, Emelia J Benjamin, Joanne M Murabito

**Affiliations:** 1 Department of Biostatistics Boston University School of Public Health Boston, MA United States; 2 Institute for Clinical Research and Health Policy Studies Tufts Medical Center Boston, MA United States; 3 Tufts Clinical and Translational Science Institute Tufts University Boston, MA United States; 4 Framingham Heart Study Boston University and National Heart, Lung, and Blood Institute Framingham, MA United States; 5 Department of Medicine University of Massachusetts Chan Medical School Worcester, MA United States; 6 Cardiology Division Department of Medicine University of Massachusetts Chan Medical School Worcester, MA United States; 7 Department of Quantitative Health Sciences University of Massachusetts Chan Medical School Worcester, MA United States; 8 CareEvolution Ann Arbor, MI United States; 9 Section of Endocrinology, Diabetes, Nutrition, and Weight Management Boston University Chobanian & Avedisian School of Medicine Boston, MA United States; 10 Center for Behavioral Science Research Department of Health Policy and Health Services Research Henry M Goldman School of Dental Medicine, Boston University Boston, MA United States; 11 Section of Cardiovascular Medicine Department of Medicine, Boston University Chobanian & Avedisian School of Medicine Boston Medical Center Boston, MA United States; 12 Department of Epidemiology Boston University School of Public Health Boston, MA United States; 13 Section of General Internal Medicine, Department of Medicine Boston University Chobanian & Avedisian School of Medicine Boston Medical Center Framingham, MA United States

**Keywords:** smartphone notifications, digital device use, randomized trial, smartphone, apps, mobile health, mHealth, devices, cardiovascular, data, intervention, blood pressure, heart rate, digital, tool, notification, messaging, prompt, nudge, behavior change, self-monitoring, self care, cardiology

## Abstract

**Background:**

Smartphone apps and mobile health devices offer innovative ways to collect longitudinal cardiovascular data. Randomized evidence regarding effective strategies to maintain longitudinal engagement is limited.

**Objective:**

This study aimed to evaluate smartphone messaging interventions on remote transmission of blood pressure (BP) and heart rate (HR) data.

**Methods:**

We conducted a 2 × 2 × 2 factorial blinded randomized trial with randomization implemented centrally to ensure allocation concealment. We invited participants from the Electronic Framingham Heart Study (eFHS), an e-cohort embedded in the FHS, and asked participants to measure their BP (Withings digital cuff) weekly and wear their smartwatch daily. We assessed 3 weekly notification strategies to promote adherence: personalized versus standard; weekend versus weekday; and morning versus evening. Personalized notifications included the participant’s name and were tailored to whether or not data from the prior week were transmitted to the research team. Intervention notification messages were delivered weekly automatically via the eFHS app. We assessed if participants transmitted at least one BP or HR measurement within 7 days of each notification after randomization. Outcomes were adherence to BP and HR transmission at 3 months (primary) and 6 months (secondary).

**Results:**

Of the 791 FHS participants, 655 (82.8%) were eligible and randomized (mean age 53, SD 9 years; 392/655, 59.8% women; 596/655, 91% White). For the personalized versus standard notifications, 38.9% (126/324) versus 28.8% (94/327) participants sent BP data at 3 months (difference=10.1%, 95% CI 2.9%-17.4%; *P*=.006), but no significant differences were observed for HR data transmission (212/324, 65.4% vs 209/327, 63.9%; *P*=.69). Personalized notifications were associated with increased BP and HR data transmission versus standard at 6 months (BP: 107/291, 36.8% vs 66/295, 22.4%; difference=14.4%, 95% CI 7.1- 21.7%; *P*<.001; HR: 186/281, 66.2% vs 158/281, 56.2%; difference=10%, 95% CI 2%-18%; *P*=.02). For BP and HR primary or secondary outcomes, there was no evidence of differences in data transmission for notifications sent on weekend versus weekday or morning versus evening.

**Conclusions:**

Personalized notifications increased longitudinal adherence to BP and HR transmission from mobile and digital devices among eFHS participants. Our results suggest that personalized messaging is a powerful tool to promote adherence to mobile health systems in cardiovascular research.

**Trial Registration:**

ClinicalTrials.gov NCT03516019; https://clinicaltrials.gov/ct2/show/NCT03516019

## Introduction

Digital technologies offer new opportunities to collect epidemiologic data and real-world evidence. Smartphones, smartwatches, and wearables have increasing penetration and use [1,2], enabling researchers to remotely collect self-reported data as well as objective measures of biometrics (eg, heart rate [HR]) or physical activity. One of the main advantages is the ability to assess participants’ behaviors outside of the clinic or research center, at repeated time points, and without recall bias.

However, participant retention and engagement is a major challenge in epidemiological and clinical trials deploying surveys or interventions with digital technologies. In the MyHeart Counts Cardiovascular Health Study, there was a rapid drop-off observed in user engagement with app use after approximately 4 days [3], and only about 10% of participants who consented to be in the study transmitted physical activity data for 7 days [4]. Similar patterns of user disengagement have been observed in other large mobile health studies [5,6]. In a large diverse data set, more than half of the participants stopped participating within the first week, and discontinuation varied by age, disease status, clinical referral, and financial incentive [7]. Although daily messaging prompts can change behavior and device use in the short term [8], robust evidence from clinical trials regarding which strategies are associated with long-term participation and use of digital technologies requires further investigation [9]. It is essential to identify interventions that can improve participant engagement in remote digital studies [7] and mitigate any concern of attrition bias to harness the potential health impacts technology can provide.

We conducted a randomized controlled trial to test the effect of smartphone app notification messaging strategies on improving participants’ long-term (3 months) use and return of HR and blood pressure (BP) data. The trial was embedded in the electronic Framingham Health Study (eFHS), an e-cohort of participants from the FHS, using a smartphone app and digital devices [10]. We hypothesized that personalized messaging compared to standard messaging would improve device use. We also tested the effect of time of day of message delivery (morning vs evening) and day of the week (weekday vs weekend) on device use. We further hypothesized that participants who are more engaged with the devices would be more likely to return the 3-month smartphone app survey.

## Methods

### Design

We conducted a 2 × 2 × 2 factorial randomized trial embedded in the eFHS. The trial was registered at ClinicalTrials.gov (NCT03516019).

### Ethics Approval

This study was approved by the Boston University Medical Campus Institutional Review Board (H-36586).

### Study Setting and Eligibility Criteria

FHS Third Generation (Gen 3; n=4095), Omni Group 2 (n=410), and New Offspring Spouse (n=103) cohorts were enrolled from 2002 to 2005 and have been examined every 6 to 8 years at the FHS research center. Participants in the eFHS were enrolled from the Gen 3, Omni Group 2, and New Offspring Spouse cohorts during the in-person examination 3 (2016-2019) at the FHS Research Center in Framingham, Massachusetts, United States [10]. eFHS eligibility criteria were as follows: English-speaking individuals who owned an iPhone (Apple Inc) with compatible iOS (version 9 or higher) or Android phone; residence in the United States; provision of permissions for notifications and data sharing with the research center; and provision of signed and dated informed consent (2-step consent as part of the research examination and within the eFHS mobile app). eFHS participants using an Android phone or who did not choose to use both devices were not eligible for the messaging trial.

At the time of the research exam, the study technician invited participants to download the eFHS app. The eFHS app allows communication with participants through notifications, permitting us to send the messaging intervention notifications defined below. Participants were asked to answer health surveys administered through the app at baseline and every 3 months. In addition, participants with iPhones were provided with choices to pair the eFHS app with a Withings-Nokia BP cuff and an Apple Watch (series 0; Apple Inc). Participants were trained in the use of the devices and asked to wear the watch daily and send a BP measurement once per week. Participants were provided with written instructions if they preferred remote enrollment. The app automatically transmits data, such as HR or BP measurements, to the FHS research center if participants have paired their devices with their iPhone.

**Figure 1:**
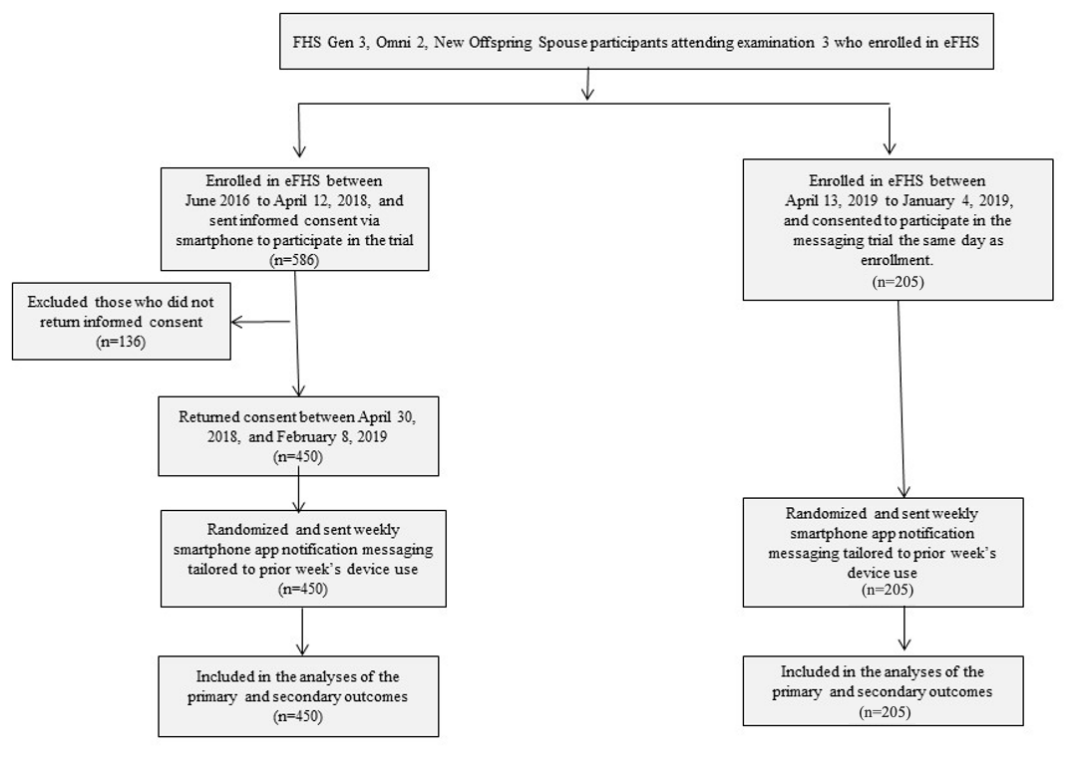
Participant flow diagram. eFHS: electronic Framingham Health Study; FHS: Framingham Health Study; Gen 3: Third Generation.

We enrolled participants in the messaging trial between April 13, 2018, and February 8, 2019, and completed the follow-up of all enrolled participants by March 23, 2019, for the primary outcome. We invited all new eFHS enrollees to participate in the messaging trial during their in-person examination 3 at the FHS research center. In addition, we contacted eligible participants who had been enrolled in the eFHS prior to the onset of the messaging trial (n=586) by email and phone to invite them to participate (Figure 1 and Multimedia Appendix 1). A consent form for participation in the trial was sent via smartphone app 1 month before trial initiation. Participants who did not return the consent survey received up to 3 reminder notifications. Participants no longer participating in the eFHS did not receive requests to consent for the messaging trial. The current trial enrolled 205 participants when they attended their in-person examination as part of the parent study and 450 participants who were previously enrolled in the eFHS.

### Interventions

#### Notifications

Prior to the current messaging trial, all eFHS participants received the following types of standard notifications through the eFHS app: (1) “Welcome to eFHS,” (2) “New surveys are available,” (3) “Reminder to complete surveys,” (4) “Surveys due,” and (5) “Thank you for completing surveys.” In addition, standard notifications were sent if no device data were received in the previous 2 weeks (Multimedia Appendix 2). Based upon our prior experience and literature review, we designed the notification messaging interventions to improve both the use of the mobile devices and response rate to surveys. Although digital interventions delivered via smartphone may be more likely to produce better engagement [11], there is limited evidence available on the characteristics influencing the effectiveness of the intervention including the timing, duration, frequency, and type of behavior change techniques [12-15]. We designed our notification messages and comparisons to address intervention characteristics that could be easily implemented and thus widely used.

**Figure 2:**
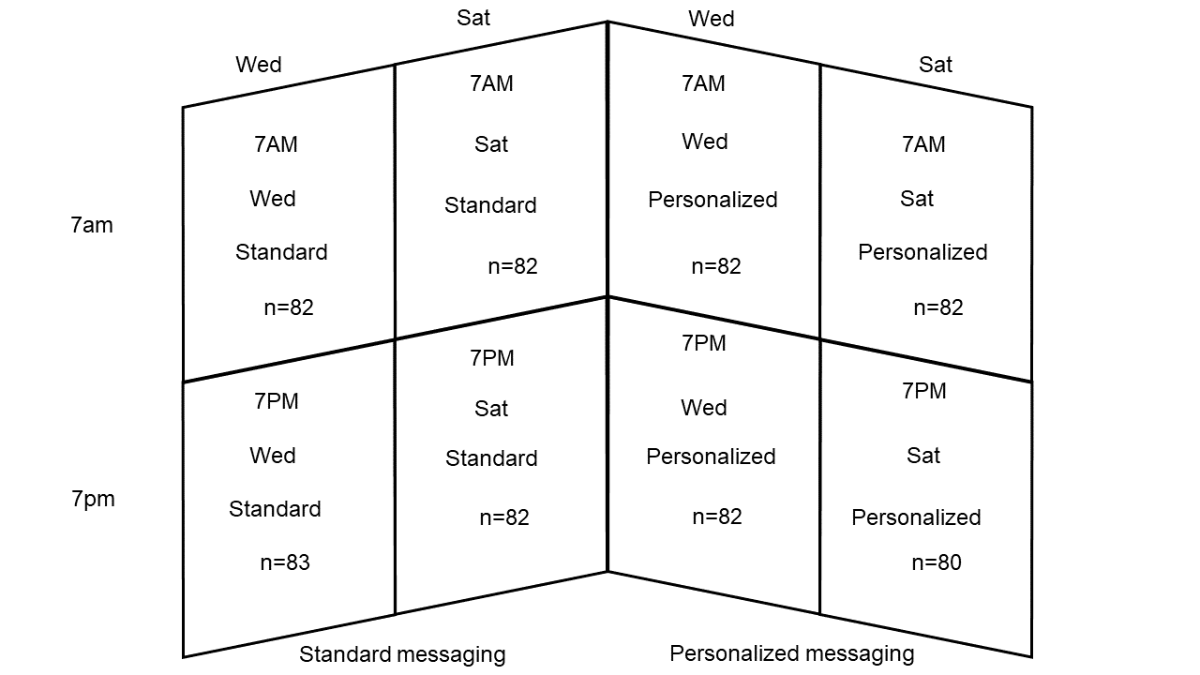
Intervention groups in the 2 × 2 × 2 factorial design of the electronic Framingham Health Study messaging trial and the number of participants. Sat: Saturday; Wed: Wednesday.

We sent weekly notifications through the app focusing on the following three comparisons: (1) personalized motivational reminder messaging versus standard messaging, (2) messaging sent on a weekend versus weekday, and (3) messaging sent in the morning versus the evening**.** We evaluated the 3 comparisons simultaneously according to a 2 × 2 × 2 factorial design. Accordingly, we randomly allocated participants to 1 of the resulting 8 groups (Figure 2). All weekly notifications were delivered automatically via the eFHS app by CareEvolution.

#### Personalization of Messaging Content

The standard notification invited participants to wear the watch daily and measure their BP weekly and was sent as a single message as follows: “We appreciate your involvement in the eFHS. Please wear your watch daily and measure your blood pressure weekly.” To personalize notifications, we included the participant’s name and customized the messaging content to whether or not the participant’s data were received in the prior week. For example, if data had not been received from the watch, the following message type was sent: “Mr X [FHS last name], we haven’t received data from your apple watch since [mm/dd/yy]. If you need help to reconnect, will you please call 508-935-xxxx or email xxxxx@bu.edu?” If data had already been received, the message was as follows: “Mr X [FHS last name] we have been receiving data from your apple watch. Keep up the good work!” One set of personalized messages targeted the use of the watch, and the other targeted the use of the BP cuff. The 2 messages (one for the watch and one for BP) were sent each week, with the message order alternating each week. Finally, to reduce notification fatigue, the specific messages were drawn at random from a library of personalized notification templates we developed with alternative motivational content (Multimedia Appendix 2). Messaging content for this trial did not address smartphone app survey return.

#### Days and Timing of Messaging

We chose specific days and times for the messaging strategies based on descriptive data collected in the eFHS between June 2016 and July 2017, reflecting the use of the devices as the day of week and time of day of digital interventions varied across published studies without a clear consensus [15-19]. We decided to test messaging sent on Wednesday (weekday) versus Saturday (weekend) and messaging sent at 7 AM (morning) versus 7 PM (evening).

### Outcomes

For the primary outcomes, we assessed if participants transmitted at least one BP or HR measurement within 7 days of each notification 3 months after randomization (after 12 notifications). In secondary analyses, we examined the transmission of BP and HR measurements at 1 month and 6 months after random allocation (after 4 and 24 notifications, respectively). We also performed a longitudinal analysis of the repeated weekly transmissions.

### Exploratory Analysis

Although the notifications did not directly encourage adherence of survey responses (they only referred to HR and BP data transmission), we hypothesized that participants who are more engaged with the devices are more likely to return the 3-month survey. As a consequence, we also examined the effect of the messaging interventions on survey completion at 3 months. We conducted this exploratory analysis only among the newly enrolled eFHS individuals in this trial, because these participants were eligible to receive the first 3-month smartphone survey. Randomization was stratified according to enrollment status (new to eFHS vs previously enrolled).

### Sample Size

Based on preliminary data from June 2016 to July 2017, the proportion of participants transmitting data at 3 months from the watch and the cuff was about 50% [10]. With a 2-sided test with an α of .05 and anticipated proportions of 50% and 70% of participants transmitting data in the groups without and with any intervention, respectively, 650 participants would give >99% power for the main effect of each intervention (Multimedia Appendix 3). This sample size assumed no interaction effect between intervention groups. We powered the trial for the three main comparisons: (1) using personalized motivational reminder messaging versus standard messaging; (2) messaging sent on a weekend versus weekday; and (3) messaging sent in the morning versus the evening, when no interaction is present. We did not adjust for multiplicity because the factorial analysis “at the margins” addresses distinct hypotheses, and statistical simulations support that no adjustment is needed in this situation [20]. If interactions existed, 650 participants also provided 77% power to detect a 2-way interaction odds ratio of 2.33 or more with a 2-sided test and an α of .05. Power to detect a 3-way interaction was limited (Multimedia Appendix 3).

### Random Allocation of Interventions

We randomly allocated each participant to 1 of 8 groups. Randomization was stratified according to sex, age (≤55 years vs >55 years), and enrollment in the eFHS prior to the beginning of the current messaging trial. A statistician (LT) generated blocked randomization lists for each stratum. Randomization was implemented centrally by CareEvolution through the eFHS app, ensuring allocation concealment. Research staff enrolled participants in the FHS research center before random allocation to one of the intervention groups, which occurred afterward. eFHS researchers, statistician, research assistant, and research center staff were masked to group assignment. As part of the eFHS, support staff helped participants complete their remote monitoring and data transmission. The support staff used standardized scripts to interact with participants over the phone. To limit the possibility of encouraging participants to use the device, we trained the support staff not to ask participants about the notification messaging content.

### Statistical Methods

Baseline characteristics of participants are presented as mean (SD) for continuous variables or percentage for categorical variables. Characteristics were obtained using standard protocols and definitions from examination 3 (Multimedia Appendix 4). We performed analyses “at the margins” to assess the main effect of each intervention, meaning that, for example, we determined the effect of personalized versus standard notifications by comparing outcomes among all participants randomly allocated to personalized notifications (regardless of days and times of messaging) with those of all participants randomly allocated to standard notifications [21]. We also evaluated 3-way and 2-way interactions between the 3 interventions (messaging content, day, and time) on the transmission of BP and HR data. For the analyses at any specific time point (1 month, 3 months, and 6 months), we used logistic regression models. We estimated the absolute difference in the proportion of participants adequately transmitting data as well as the odds ratio. For the longitudinal analysis of the repeated weekly transmissions, we used a logistic regression model with random intercept. For the analysis of 3-month survey completion, we defined complete, partial, and missing response as all, some, or no survey questions answered. We performed this analysis in the subgroup of individuals who had not been enrolled in the eFHS prior to the beginning of the messaging trial. We used chi-square tests to compare the proportion of complete, partial, and missing response between intervention groups “at the margins.” Two-sided tests were performed for all analyses with a significance level of .05.

## Results

### Participant Characteristics

Of the 791 eligible FHS participants, we received consent from and randomly allocated 655 (82.8%) participants (Figure 1). Table 1 summarizes participant characteristics. Participants were, on average, aged 53 (SD 9) years, and 59.8% (392/655) were women. In all, 450 participants had already been enrolled in the eFHS prior to the beginning of the randomized messaging trial, including 232 participants aged >55 years, and 205 participants were new eFHS participants, including 69 participants aged >55 years. Characteristics of participants in the trial were similar to the eFHS participants who chose to use BP cuffs or smartwatches but were not part of the trial (Multimedia Appendix 5). Overall, the proportion of participants transmitting BP and HR measurements decreased over time. HR transmission via the smartwatch was higher than BP transmission. Participants aged >55 years were more likely to transmit BP and HR measurements (Multimedia Appendices 6 and 7). At 1, 3, and 6 months after randomization, 130/300 (43.3%), 129/298 (43.3%), and 110/270 (40.7%) of participants aged >55 years transmitted BP data compared to 119/353 (33.7%), 91/353 (25.8%), and 63/316 (19.9%) of participants ≤55 years, respectively (longitudinal model *P* value <.001); 205/300 (68.3%), 205/298 (68.8%), 170/258 (65.9%) of participants aged >55 years transmitted HR data compared to 237/363 (65.3%), 216/353 (61.2%), and 174/304 (57.2%) of participants ≤55 years, respectively (longitudinal model *P* value <.001).

**Table 1:** Characteristics of the 655 participants^a^ enrolled in the electronic Framingham Health Study (eFHS) messaging trial 2018-2019.

Variable	Value (N=655)
Age (years), mean (SD)	53 (9)
Age, >55 years, n (%)	301 (46)
Women, n (%)	392 (59.8)
Multiethnic/multiracial Omni group 2 participants, n (%)	59 (9)
Body mass index (kg/m^2^), mean (SD)	28 (5)
Systolic BP (mmHg), mean (SD)	119 (14)
Diastolic BP (mmHg), mean (SD)	76 (8)
Physical activity index, mean (SD)	33 (5)
Current smoking, n (%)	32 (4.9)
Diabetes, n (%)	40 (6.1)
Hypertension, n (%)	185 (28.2)
Prior cardiovascular disease, n (%)	27 (4.1)

^a^In all, 450 participants had already been enrolled in the eFHS prior to the beginning of the randomized messaging trial, and 205 participants were new eFHS participants. Physical activity was assessed using the FHS physical activity index, a composite score based on the number of hours spent sleeping or in sedentary, slight, moderate, and heavy activities during a 24-hour period. Weights of 1, 1.1, 1.5, 2.4, and 5 were assigned to sleep, sedentary, slight, moderate, and heavy activity, respectively.

### Effect of Interventions on the Transmission of BP Measurements

At 3 months after randomization, the proportion of participants who transmitted BP measurements was larger in the personalized messaging group compared to the standard messaging group (126/324, 38.9% vs 94/327, 28.8%), for an absolute difference of 10.1% (95% CI 2.9%-17.4%; *P*=.006; Table 2). There was no evidence of a difference in the proportions of participants transmitting BP measurements between messaging sent on a weekend versus weekday (111/324, 34.3% vs 109/327, 33.3%; *P*=.80) or between messaging sent in the morning (7 AM) versus the evening (7 PM; 114/326, 35% vs 106/325, 32.6%; *P*=.53) at 3 months. Similar results were observed at 1 month and 6 months (Table 2).

There was no evidence of interaction between the 3 types of messaging interventions (personalized vs standard, weekend vs weekday, and morning vs evening; Multimedia Appendices 8 and 9). We observed an odds ratio of 2.03 (95% CI 1.27-3.24) versus 1.24 (95% CI 0.78-1.96) for personalized versus standard notification when notifications were sent during the week as opposed to the weekend, which was not statistically significant (interaction *P*=.14; Multimedia Appendix 9).

**Figure 3:**
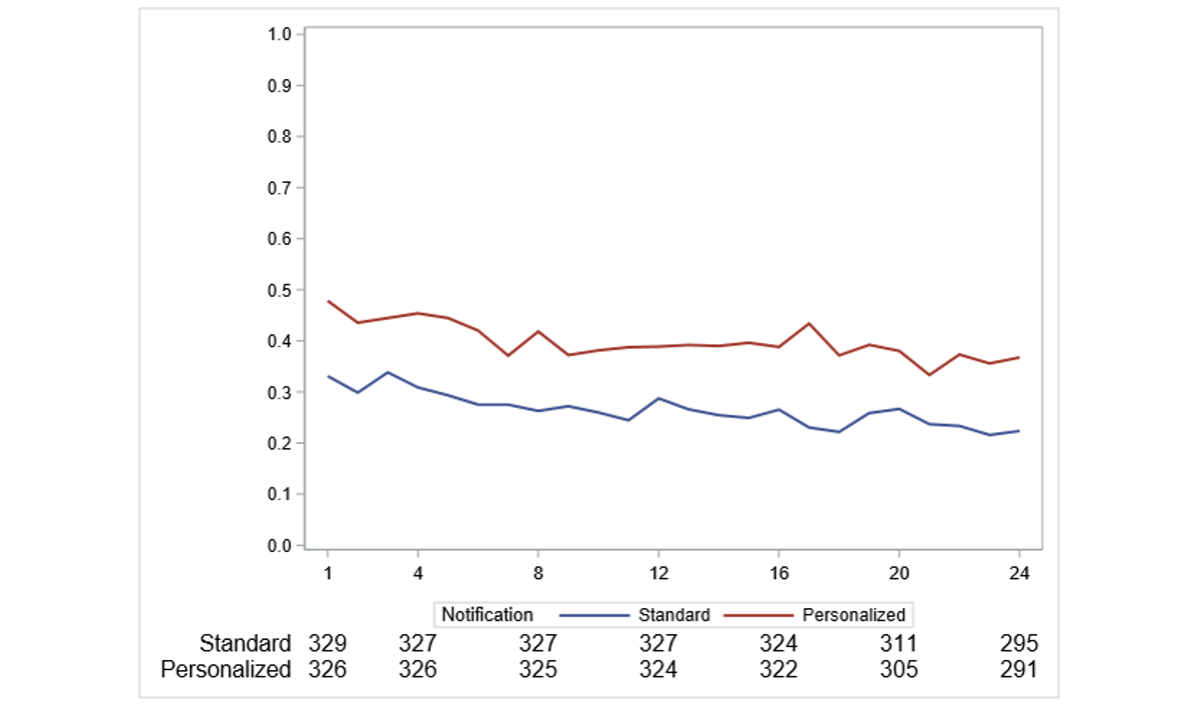
Proportion of participants transmitting at least one blood pressure measurement within 7 days of each weekly notification in the personalized versus standard messaging groups.

In the longitudinal analysis of weekly transmission, there was also evidence that the proportion of participants who transmitted BP measurements was larger in the personalized messaging group compared to the standard messaging group over 24 weeks (*P*=.04; Figure 3 and Multimedia Appendix 10). Notifications sent in the morning were associated with slightly increased BP transmission over 24 weeks as compared to evening notifications (*P*=.03; Multimedia Appendix 11). There was no evidence of a difference over time for weekend versus weekday (*P*=.80; Multimedia Appendix 12).

**Table 2:** Comparisons of proportions of participants transmitting blood pressure measurements according to messaging trial assignment.

Comparison, time after random allocation		Proportion transmitting, n/N (%)		Odds ratio (95% CI)	Difference in proportions (%; 95% CI)	*P* value
		Intervention group	Nonintervention group			
**Personalized messaging (n=326) vs standard neutral messaging (n=329)**						
	1 month	148/326 (45.4)	101/327 (30.9)	1.86 (1.35 to 2.56)	14.5 (7.1 to 21.9)	<.001
	3 months	126/324 (38.9)	94/327 (28.8)	1.58 (1.14 to 2.19)	10.1 (2.9 to17.4)	.006
	6 months	107/291 (36.8)	66/295 (22.4)	2.02 (1.40 to 2.90)	14.4 (7.1 to 21.7)	<.001
**Messaging sent in the morning (n=328) vs the evening (n=327)**						
	1 month	130/326 (39.9)	119/327 (36.4)	1.16 (0.85 to 1.59)	3.5 (–4.0 to 10.9)	.36
	3 months	114/326 (35)	106/325 (32.6)	1.11 (0.80 to 1.54)	2.4 (–4.9 to 9.6)	.53
	6 months	93/293 (31.7)	80/293 (27.3)	1.24 (0.87 to 1.77)	4.4 (–2.9 to 11.8)	.24
**Messaging sent on a weekend (n=326) vs weekday (n=329)**						
	1 month	125/325 (38.5)	124/328 (37.8)	1.03 (0.75 to 1.41)	0.7 (–6.8 to 8.1)	.86
	3 months	111/324 (34.3)	109/327 (33.3)	1.04 (0.75 to 1.44)	0.9 (–6.3 to 8.2)	.80
	6 months	92/289 (31.8)	81/297 (27.3)	1.25 (0.87 to 1.78)	4.6 (–2.8 to 11.9)	.23

### Effect of Interventions on the Transmission of HR Measurements

At 3 months after randomization, there was no evidence of a difference in the proportions of participants transmitting HR measurements for any of the intervention: personalized versus standard notification (212/324, 65.4% vs 209/327, 63.9%; *P*=.69), morning versus evening notifications (212/326, 65% vs 209/325, 64.3%; *P*=.85), and weekend versus weekday notifications (210/324, 64.8% vs 211/327, 64.5%; *P*=.94). Results at 1 month and 6 months are shown in Table 3. The proportions of participants transmitting HR was higher in the personalized versus standard notification (186/281, 66.2% vs 158/281, 56.2%; absolute difference=10%, 95% CI 2%-18%; *P*=.02).

**Figure 4:**
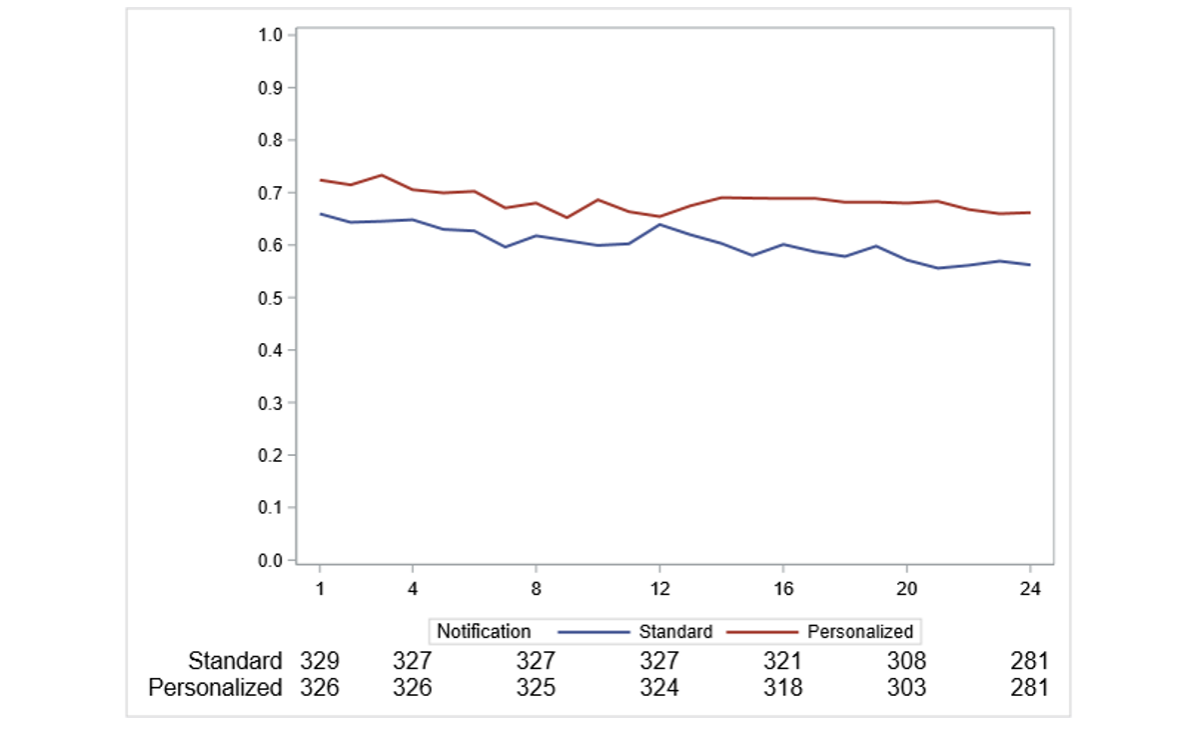
Proportion of participants transmitting at least one heart rate measurement within 7 days of each weekly notification in the personalized versus standard messaging groups.

There was no evidence of interaction between interventions (Multimedia Appendices 13 and 14). In the longitudinal analysis leveraging 24 weeks of weekly messages, the proportion of participants who transmitted HR measurements was larger in the personalized messaging group compared to the standard messaging group over 24 weeks (*P*=.02; Figure 4 and Multimedia Appendix 15). There was no evidence of a difference over time for morning versus evening notifications (*P*=.54; Multimedia Appendix 16) and weekend versus weekday notifications (*P*=.90; Multimedia Appendix 17).

**Table 3:** Comparisons of proportions of participants transmitting heart rate measurements according to messaging trial assignment.

Comparison, time after random allocation			Proportion transmitting, n (%)			Odds ratio (95% CI)		Difference in proportions (%; 95% CI)		*P* value
		Intervention group		Nonintervention group						
**Personalized messaging (n=326) vs standard neutral messaging (n=329)**										
	1 month	230/326 (70.6)		212/327 (64.8)	1.30 (0.94 to 1.81)		5.7 (–1.4 to 12.9)		.12	
	3 months	212/324 (65.4)		209/327 (63.9)	1.07 (0.78 to 1.47)		1.5 (–5.8 to 8.9)		.69	
	6 months	186/281 (66.2)		158/281 (56.2)	1.52 (1.08 to 2.15)		10 (2.0 to 18.0)		.02	
**Messaging sent in the morning (n=328) vs the evening (n=327)**										
	1 month	231/326 (70.9)		212/327 (64.8)	1.34 (0.96 to 1.86)		6.3 (–0.8 to 13.5)		.08	
	3 months	212/326 (65)		209/325 (64.3)	1.03 (0.75 to 1.42)		0.7 (–8.1 to 6.6)		.85	
	6 months	181/280 (64.6)		163/282 (57.8)	1.34 (0.95 to 1.88)		6.8 (–1.2 to 14.9)		.10	
**Messaging sent on weekend (n=326) vs weekday (n=329)**										
	1 month	228/325 (70.2)		214/328 (65.2)	1.25 (0.90 to 1.74)		4.9 (–2.3 to 12.1)		.18	
	3 months	210/324 (64.8)		211/327 (64.5)	1.01 (0.73 to 1.40)		0.3 (–7.1 to 7.6)		.94	
	6 months	179/276 (64.9)		165/286 (57.7)	1.35 (0.96 to 1.90)		7.2 (–0.9 to 15.2)		.08	

### Effects of Interventions on Survey Completion

Among 205 participants who had not been enrolled in the eFHS prior to the beginning of the messaging trial, there was no evidence of a difference between intervention groups in terms of survey completion. In the personalized notification group, 58.8% (60/102) and 41.2% (42/102) of participants had complete and missing survey response at 3 months, respectively, as opposed to 57.3% (59/103) and 42.7% (44/103) in the standard notification group (*P*=.83; Multimedia Appendix 18).

## Discussion

We evaluated 3 smartphone app notification intervention strategies sent to eFHS participants in a randomized trial aimed at improving the use of a smartwatch and a digital BP cuff for the remote monitoring of HR and BP. We observed evidence that personalized motivational notifications increased adherence over 3 months for BP data transmission and over 6 months for BP and HR transmission. Personalization included the participant’s name, and the message content was contextualized to the individual’s device use for each week of the trial. The use of the smartwatch and the BP cuff decreased over time, but the proportions of participants transmitting BP and HR data were about 10% higher in the personalized versus standard notification groups. There was little or no evidence that morning versus evening notifications or weekend versus weekday notifications were associated with adherence. In an exploratory aim, we did not observe a difference in smartphone app survey completion between intervention groups, but the analysis may have been underpowered. This trial provides evidence that short personalized motivational messaging offers an easy, scalable strategy to increase adherence with device use that potentially can be used in other large digital intervention studies.

The investigation of a diverse set of digital health studies, including a range of diseases in more than 100,000 participants, revealed early user disengagement with a median study retention across all studies of fewer than 6 days [7]. Despite this finding, few studies have explored digital messaging interventions to improve adherence to digital device use in large samples [9]. Increasing evidence supports the benefit brief messaging such as SMS text messaging to enhance self-care in the management of cardiovascular disease risk factors including hypertension [15,22]. The use of SMS text messaging can be effective in promoting accelerometer use, with wear hours observed to be longer in participants receiving messaging versus those not receiving messaging [23]. However, in many prior studies, messaging content has largely not been personalized. Our trial provides evidence of the effectiveness of personalized notification strategies on enhancing device use, addressing a critical knowledge gap given the limited evidence available on the effectiveness of digital strategies to enhance the retention and engagement of participants in e-cohorts and clinical trials [24].

We observed that compared to younger participants, older participants were more likely to transmit BP and HR data. This finding appears to be consistent with another study that reported older age was associated with retention time [7]. Although this observation may be at odds with overall lower technology use and digital literacy among older adults [25], in our study, smartphone ownership was an eligibility criterion, raising the possibility that older participants enrolled in this trial were more comfortable with technology. In addition, having the condition under study may enhance retention [7]. Although participants in the eFHS were not enrolled for any particular medical condition, hypertension and cardiovascular risk factors and disease increase in prevalence with advancing age. Therefore, it is possible older adults were more interested in monitoring BP and HR.

Our trial used weekly notifications to enhance the use of digital devices over 6 months to monitor BP and HR. We chose a weekly delivery to limit participant burden and associated notification fatigue observed in other trials. However, future investigations are needed to examine the intensity of notification delivery to determine whether more frequent notifications would result in higher observed device use.

Our trial did not find any clear evidence for an effect of the day of the week or time of day on device use. In contrast, a trial of the effect of notification messaging on engagement with a wellness app showed that the effect was greatest when delivered on the weekend at midday [26]. Whether providing participants the opportunity to choose a notification delivery day and time that they perceive as convenient enhances engagement and user perception that the notifications are helpful remains to be studied [27].

Notification content that includes multiple behavior change strategies [28] and novel adaptive designs that are dynamic and customized to the individual have the potential to be even more effective in changing behaviors and thus enhancing engagement [29]. More recently, reinforcement learning, a machine learning technique, has been used to optimize personalized approaches to messaging [30]. Further examination of how these strategies can be applied to decrease attrition and increase adherence to digital study protocols is needed.

We embedded this trial in the eFHS, which provides several strengths. FHS participants have been enrolled in the parent study for over a decade (first examination from 2002-2005), demonstrate loyalty to the parent study; the participants have developed trust in parent study investigators and staff that may have facilitated higher levels of retention and engagement than seen in other e-cohorts [4]. eFHS participants are well characterized with directly measured BP and cardiovascular risk factors at in-person research center examinations.

Our study also has some limitations that merit comment. We did not adjust for multiplicity related to coprimary BP and HR outcomes. We could have considered a multiplicity testing procedure, such the Holm procedure, to preserve the family-wise error rate and calculated the required sample size for the simple disjunctive power [31]. In retrospect, our conclusions would be similar. Participants were generally healthier than the full FHS cohorts and not recruited for any specific health condition. Personalized notifications may have resulted in greater observed differences in HR and BP transmission in persons with underlying hypertension and cardiovascular disease. Our sample participants were primarily White, well educated, and residing in New England. Thus, our results may not be generalizable to persons of diverse racial or ethnic backgrounds, other regions, or those with different measures of social determinants of health or levels of digital literacy. Our trial was conducted prior to the COVID-19 pandemic. Many clinical trials replaced in-person visits with remote data collection, and personalized notifications may help reducing drop-out in decentralized trials [32]. Finally, we could not confirm that the notification messages were actually read by participants.

Brief nonobtrusive personalized motivational smartphone app notifications delivered once per week significantly increased the use of a smartwatch and wireless BP device in middle-aged to older adults for monitoring cardiovascular measures (HR and BP) over 6 months. These simple prompts can be easily incorporated and scaled by other digital health studies to promote long-term engagement. Larger studies of more diverse participants are needed, as well as further study of the impact of notification strategies for digital and mobile health monitoring on health outcomes.

## Appendix

Multimedia Appendix 1 Script for contacting previously enrolled electronic Framingham Health Study participants to join the messaging trial.

Multimedia Appendix 2 Library of messages used in the trial.

Multimedia Appendix 3 Power calculations.

Multimedia Appendix 4 Participant characteristic definitions.

Multimedia Appendix 5 Characteristics of electronic Framingham Health Study participants using a blood pressure cuff or smartwatch not participating in the trial.

Multimedia Appendix 6 Proportion of participants transmitting at least one blood pressure measurement within 7 days of each weekly notification according to age and electronic Framingham Health Study enrollment status.

Multimedia Appendix 7 Proportion of participants transmitting at least one heart rate measurement within 7 days of each weekly notification according to age and electronic Framingham Health Study enrollment status.

Multimedia Appendix 8 Three-way and two-way interaction analyses for the proportion of participants transmitting at least one blood pressure measurement within 7 days of each weekly notification.

Multimedia Appendix 9 Odds ratios from the two-way interaction analyses for the proportion of participants transmitting at least one blood pressure measurement within 7 days of each weekly notification.

Multimedia Appendix 10 Longitudinal analysis of the weekly proportion of participants transmitting at least one blood pressure measurement within 7 days of each weekly notification.

Multimedia Appendix 11 Proportion of participants transmitting at least one blood pressure measurement within 7 days of each weekly notification in the morning and evening notification groups.

Multimedia Appendix 12 Proportion of participants transmitting at least one blood pressure measurement within 7 days of each weekly notification in the weekend and weekday notification groups.

Multimedia Appendix 13 Three-way and two-way interaction analyses for the proportion of participants transmitting at least one heart rate measurement within 7 days of each weekly notification.

Multimedia Appendix 14 Odds ratios from the two-way interaction analyses for the proportion of participants transmitting at least one heart rate measurement within 7 days of each weekly notification.

Multimedia Appendix 15 Longitudinal analysis of the weekly proportion of participants transmitting at least one heart rate measurement within 7 days of each weekly notification.

Multimedia Appendix 16 Proportion of participants transmitting at least one heart rate measurement within 7 days of each weekly notification in the morning and evening notification groups.

Multimedia Appendix 17 Proportion of participants transmitting at least one heart rate measurement within 7 days of each weekly notification in the weekend and weekday notification groups.

Multimedia Appendix 18 Survey completion among 205 individuals who had not been enrolled in the electronic Framingham Health Study prior to the beginning of the messaging trial.

Multimedia Appendix 19 CONSORT-eHEALTH checklist (V 1.6.1).

## Abbreviations

BP: blood pressure
eFHS: electronic Framingham Heart Study
FHS: Framingham Heart Study
Gen 3: Third Generation
HR: heart rate

## Data Availability

Electronic Framingham Health Study (eFHS) data are available from the FHS and will be deposited in the National Heart, Lung, and Blood Institute (NHLBI) Biologic Specimen and Data Repository Information Coordinating Center (BioLINCC) for data sharing.
