# Supplementary material for: Increasing Engagement in the Electronic Framingham Heart Study: Factorial Randomized Controlled Trial
Source: J Med Internet Res. 2023 Jan 20;25:e40784. doi: 10.2196/40784 (PMC9898831; doi:10.2196/40784)
Supplement: Multimedia Appendix 1 [file jmir_v25i1e40784_app1.docx]

# Multimedia Appendix 1. Script for contacting previously enrolled eFHS participants to join the messaging trial

**Messages used in eFHS prior to RCT**

Welcome to eFHS: Welcome to the electronic Framingham Heart Study. During the next two weeks, please complete your initial surveys available on the “surveys” tab below.

New surveys Available: There are new surveys available to complete. Please open the eFHS app and complete them.

Survey due in 1 week: Reminder: You have surveys to complete

Survey due today: You have surveys due today. Please open the eFHS app and complete them.

No data from BP cuff or from Apple Watch in last 2 weeks:

We have not received a blood pressure reading from you in over 2 weeks. Please take your blood pressure on a weekly basis. Your continued participation is critical, call us at 508-935-3xxx if you have questions or issues.

We have not received a Heart Rate reading from your Apple Watch in over 2 weeks. Please continue wearing it. Your continued participation is critical, call us at 508-935-3xxx if you have any questions or issues.
