# Supplementary material for: Increasing Engagement in the Electronic Framingham Heart Study: Factorial Randomized Controlled Trial
Source: J Med Internet Res. 2023 Jan 20;25:e40784. doi: 10.2196/40784 (PMC9898831; doi:10.2196/40784)
Supplement: Multimedia Appendix 2 [file jmir_v25i1e40784_app2.docx]

# Multimedia Appendix 2. Notifications

**Library of messages used in RCT**

Standard message

“We appreciate your involvement in the eFHS. Please wear your watch daily and measure your blood pressure weekly.”

Personalized messages

| Blood Pressures |
| --- |
| 1) Use of BP cuff |
| A) “Mr. (S) [FHS last name], we received your blood pressure last week. Thank you and keep it up!” |
| B) “Mr. (S) [FHS last name], way to go! We received another blood pressure from you last week. Thank you!” |
| C) “Mr. (S) [FHS last name], our team is grateful for another blood pressure upload. Nice job!” |
| D) ““Mr. (S) [FHS last name], we got your blood pressure reading! You are on a roll! Keep it up.” |
| E) “Ms. (r) [FHS last name], Most excellent! The eFHS team received your blood pressure last week. |
| 2) No Use of BP cuff |
| A) Mr. (S) [FHS last name], we missed your blood pressure last week. We would really appreciate it if you could send us another reading.” |
| B) “Mr. (S) [FHS last name], say it isn’t so! We hope we do not go another week without a blood pressure from you!” |
| C) “Mr. (S) [FHS last name], come back! We miss getting your weekly blood pressure readings!” |
| D) ““Mr. (S) [FHS last name], we know you are busy but we miss hearing from you! Please send us your blood pressure!” |
| E) “Ms. (r) [FHS last name], we missed receiving your blood pressure last week. Kelsey at 508-935-xxxx or xxxxxx@bu.edu will enthusiastically help you troubleshoot any issues with your device." |

| Watches |
| --- |
| 1) Use of watch |
| A) "Mr. (S) [FHS last name], our team is grateful that you have been wearing your apple watch! Your data will be used in important FHS research!" |
| B) "Mr. (S) [FHS last name], We are writing to let you know that we have been receiving data from your apple watch. Thank you for your continued contributions to our eFHS project! |
| C) "Mr. (S) [FHS last name], we have been receiving data from your apple watch. Keep up the good work!" |
| D) "Mr. (S) [FHS last name], we know you are busy and we thank you for taking the time to participate in our eFHS study. We have been receiving data from your apple watch. Keep up the good work!" |
| E) “Ms. (r) [FHS last name], way to go! We greatly appreciate receiving your apple watch data last week. |
| F) “Ms. (r) [FHS last name], We received your apple watch data last week. Please keep up the great work! |
| G) “Ms. (r) [FHS last name], Your last week’s apple watch readings came in on schedule. Thank you for providing valuable data to eFHS! |
| H) “Ms. (r) [FHS last name], Kudos to you for using your apple watch last week! Appreciatively, the eFHS research team. |
| 2) No Use of Watch |
| A) "Mr. (S) [FHS last name], we have not been receiving data from your apple watch. If you need assistance please contact Emily or Kelsey at xxx-xxx-xxxx." |
| B) "Mr. (S) [FHS last name], we have not been receiving your apple watch data recently. We know that you are busy but please try to wear your watch soon!" |
| C) "Mr. (S) [FHS last name], we miss you! We have not received data from your apple watch since (mm/dd/yy). Please call 508-935-xxxx or email kmfusco@bu.edu if you are having any trouble!" |
| D) "Mr. (S) [FHS last name], we have not received data from your apple watch in some time. Please wear your watch when possible, your data are important to ongoing FHS research!" |
| E) "Mr. (S) [FHS last name], we have not received data from your apple watch since (mm/dd/yy). Please make sure that your eFHS app is open and that your phone is connected to wifi." |
| F) "Mr. (S) [FHS last name], we haven’t received data from your apple watch since (mm/dd/yy). If you need help to reconnect, will you please call 508-935-xxxx or email xxxxxxx@bu.edu?" |
| G) "Mr. (S) [FHS last name], we hope all is well. We haven’t received your apple watch data since (mm/dd/yy). May we please help you reconnect? Kelsey at 508-935-xxxx or xxxxxxx@bu.edu is happy to troubleshoot any issues." |
| H) "Mr. (S) [FHS last name], we haven’t received data from your apple watch since (mm/dd/yy). May we help you with the apple watch? Kelsey is available to help at 508-935-xxxx or email xxxxxxx@bu.edu" |
