# Supplementary material for: Increasing Engagement in the Electronic Framingham Heart Study: Factorial Randomized Controlled Trial
Source: J Med Internet Res. 2023 Jan 20;25:e40784. doi: 10.2196/40784 (PMC9898831; doi:10.2196/40784)
Supplement: Multimedia Appendix 3 [file jmir_v25i1e40784_app3.docx]

# Multimedia Appendix 3 – Power calculations

We code the levels of covariates by -1 (no) and +1 (yes) such that the linear predictor of the 2x2x2 factorial model with an interaction has the form Y=β_0_+ β_1_x_1_+ β_2_x_2_+ β_3_ x_3_+ β_4_ x_1_x_2_+ β_5_ x_1_x_3_+ β_6_ x_2_x_3_+ β_7_ x_1_x_2_x_3_

| X1 | X2 | X3 | Y |
| --- | --- | --- | --- |
| -1 | -1 | -1 | β_0_- β_1_- β_2_- β_3_ + β_4_ + β_5_ + β_6_ - β_7_ |
| -1 | -1 | 1 | β_0_- β_1_- β_2_+ β_3_ + β_4_ - β_5_ - β_6_ + β_7_ |
| -1 | 1 | -1 | β_0_- β_1_+ β_2_- β_3_ - β_4_ + β_5_ - β_6_ + β_7_ |
| -1 | 1 | 1 | β_0_- β_1_+ β_2_+ β_3_ - β_4_ - β_5_ + β_6_ - β_7_ |
| 1 | -1 | -1 | β_0_+ β_1_- β_2_- β_3_ - β_4_ - β_5_ + β_6_ + β_7_ |
| 1 | -1 | 1 | β_0_+ β_1_- β_2_+ β_3_ - β_4_ + β_5_ - β_6_ - β_7_ |
| 1 | 1 | -1 | β_0_+ β_1_+ β_2_- β_3_ + β_4_ - β_5_ - β_6_ - β_7_ |
| 1 | 1 | 1 | β_0_+ β_1_+ β_2_+ β_3_ + β_4_ + β_5_ + β_6_ + β_7_ |

The main effects and interaction terms are related to the parameters as defined in the table below. Parameter estimates correspond to log-odds ratios. N refers to the total sample size of the trial, p to the average probability of data transmission.

| Effect |  |  | Approximate variance |
| --- | --- | --- | --- |
| A | 0.5(Y_1.._-Y_-1.._) | 2β_1_ | 4/(Np(1-p)) |
| B | 0.5(Y_.1._-Y_.-1._) | 2β_2_ | 4/(Np(1-p)) |
| C | 0.5(Y_..1_-Y_..-1_) | 2β_3_ | 4/(Np(1-p)) |
| A:B | 0.25[(Y_11._-Y_-11._)- (Y_1-1._-Y_-1-1._)] | β_1_/2-β_2_/2+β_4_ | 3/(2Np(1-p)) |
| A:C | 0.25[(Y_.11_-Y_.-11_)- (Y_.1-1_-Y_.-1-1_)] | β_1_/2-β_3_/2+β_4_ | 3/(2Np(1-p)) |
| B:C | 0.25[(Y_1.1_-Y_-1.1_)- (Y_1.-1_-Y_-1.-1_)] | β_2_/2-β_3_/2+β_4_ | 3/(2Np(1-p)) |
| A:B:C | 0.125{[(Y_11-1_-Y_-11-1_)- (Y_1-1-1_-Y_-1-1-1_)]- [(Y_111_-Y_-111_)- (Y_1-11_-Y_-1-11_)]} | β_7_ | 1/(Np(1-p)) |

Based on the target value for a main effect δ and its approximate standard error from the table above, the approximate power based of a two-sided test at level α/2 can then be determined as

Power=1-Φ(z_1-α/2_- δ/se )+ Φ(- z_1-α/2_- δ/se)

where z1-α/2 and Φ corresponds to the quantile and the cumulative distribution function of the standard normal distribution. Power calculations for main effects, 2-way interaction, and 3-way interaction are reported below.

| P0 | P1 | OR | Power  main effect | Power  2-way  interaction | Power  3-way  interaction |
| --- | --- | --- | --- | --- | --- |
| 0.4 | 0.5 | 1.50 | 0.716 | 0.450 | 0.097 |
| 0.4 | 0.6 | 2.25 | 0.999 | 0.716 | 0.245 |
| 0.4 | 0.65 | 2.79 | 1 | 0.892 | 0.359 |
| 0.4 | 0.7 | 3.50 | 1 | 0.9745 | 0.498 |
| 0.5 | 0.6 | 1.50 | 0.734 | 0.253 | 0.099 |
| 0.5 | 0.7 | 2.33 | 1 | 0.770 | 0.271 |
| 0.5 | 0.75 | 3.00 | 1 | 0.938 | 0.417 |
| 0.5 | 0.8 | 4.00 | 1 | 0.993 | 0.598 |
| 0.6 | 0.7 | 1.56 | 0.789 | 0.281 | 0.106 |
| 0.6 | 0.8 | 2.67 | 1 | 0.865 | 0.334 |
| 0.6 | 0.85 | 3.78 | 1 | 0.986 | 0.546 |
| 0.6 | 0.9 | 6.00 | 1 | 1 | 0.799 |
