# Supplementary material for: Increasing Engagement in the Electronic Framingham Heart Study: Factorial Randomized Controlled Trial
Source: J Med Internet Res. 2023 Jan 20;25:e40784. doi: 10.2196/40784 (PMC9898831; doi:10.2196/40784)
Supplement: Multimedia Appendix 4 [file jmir_v25i1e40784_app4.docx]

# Multimedia Appendix 4. Participant characteristic definitions

Participant characteristics were obtained using data from FHS exam 3 (time of enrollment to eFHS). Height and weight were obtained by trained technicians and body mass index was calculated by dividing the participant’s weight in kilograms by the square of the height in meters (kg/m^2^). Current smoking was defined as self-report of smoking in the year prior to the examination. Systolic and diastolic blood pressure was the average of two resting blood pressure measurements. Physical activity was assessed using the FHS physical activity index (PAI) a composite score based on the number of hours spent sleeping, or in sedentary, slight, moderate, and heavy activities during a 24-hour period. Weights of 1, 1.1, 1.5, 2.4 and 5 were assigned to sleep, sedentary, slight, moderate and heavy activity, respectively as previously reported. Hypertension was defined as the average of two resting blood pressure measurements of ≥140/90 mmHg or report of antihypertensive medication use. Type 2 diabetes was defined as fasting glucose ≥126 mg/dl or report of use of hypoglycemic medication. Prevalent cardiovascular disease included coronary heart disease (myocardial infarction, angina pectoris, coronary insufficiency), stroke, intermittent claudication, and heart failure, after adjudication by a panel of senior investigators using standard criteria and all available information including hospital records.
