# Supplementary material for: Increasing Engagement in the Electronic Framingham Heart Study: Factorial Randomized Controlled Trial
Source: J Med Internet Res. 2023 Jan 20;25:e40784. doi: 10.2196/40784 (PMC9898831; doi:10.2196/40784)
Supplement: Multimedia Appendix 5 [file jmir_v25i1e40784_app5.docx]

# Multimedia Appendix 5: Table S1. Characteristics of eFHS participants using a BP cuff and or smartwatch not participating in the RCT

| Variable | N=731 |
| --- | --- |
| Age, years | 53±9 |
| ≥55 years | 41% |
| Women | 59% |
| Multi-ethnic Omni participants | 8% |
| Body mass index, kg/m2 | 29±6 |
| Systolic BP, mmHg | 118±14 |
| Diastolic BP, mmHg | 76±8 |
| Physical Activity Index | 34±5 |
| Current smokers | 5% |
| Diabetes | 5% |
| Hypertension | 24% |
| Prior cardiovascular disease | 3% |
