# Supplementary material for: Increasing Engagement in the Electronic Framingham Heart Study: Factorial Randomized Controlled Trial
Source: J Med Internet Res. 2023 Jan 20;25:e40784. doi: 10.2196/40784 (PMC9898831; doi:10.2196/40784)
Supplement: Multimedia Appendix 6 [file jmir_v25i1e40784_app6.docx]

**Multimedia Appendix 6. Figure S1. Proportion of participants transmitting at least one BP measurement within 7 days of each weekly notification according to age and eFHS enrollment status**


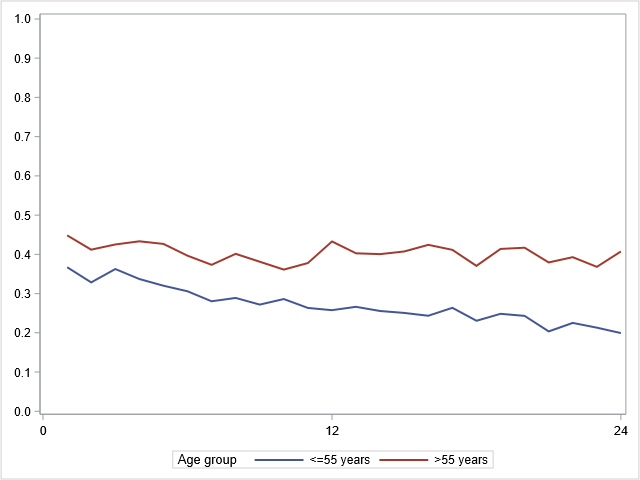


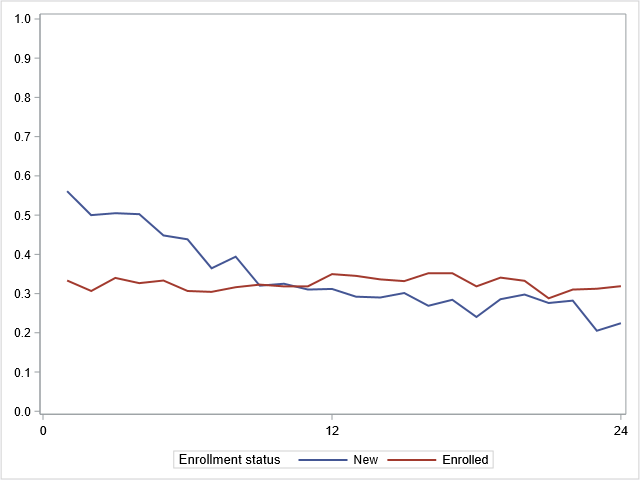


Enrolled: already enrolled in eFHS prior to the beginning of the randomized messaging trial; New: new eFHS enrollee.
