# Supplementary material for: Increasing Engagement in the Electronic Framingham Heart Study: Factorial Randomized Controlled Trial
Source: J Med Internet Res. 2023 Jan 20;25:e40784. doi: 10.2196/40784 (PMC9898831; doi:10.2196/40784)
Supplement: Multimedia Appendix 8 [file jmir_v25i1e40784_app8.docx]

# Multimedia Appendix 8: Table S2. Three-way and two-way interaction analyses for the proportion of participants transmitting at least one BP measurement within 7 days of each weekly notification

|  | β | SE | p |
| --- | --- | --- | --- |
| *Three-way interaction* |  |  |  |
| intercept | -1.0197 | 0.2487 | <.0001 |
| weekend | 0.0777 | 0.3497 | 0.8241 |
| am | -0.0951 | 0.3581 | 0.7906 |
| weekend*am | 0.4532 | 0.4923 | 0.3573 |
| personalized | 0.6450 | 0.3361 | 0.0550 |
| weekend*personalized | -0.3027 | 0.4783 | 0.5268 |
| am* personalized | 0.1250 | 0.4792 | 0.7943 |
| weekend*am* personalized | -0.3812 | 0.6715 | 0.5702 |
| *Two-way interaction 1* | β | SE | p |
| intercept | -0.6840 | 0.1654 | <.0001 |
| weekend | -0.0846 | 0.2368 | 0.7207 |
| am | -0.0183 | 0.2346 | 0.9377 |
| weekend*am | 0.2466 | 0.3318 | 0.4574 |
| *Two-way interaction 2* | β | SE | p |
| intercept | -1.0662 | 0.1789 | <.0001 |
| weekend | 0.3079 | 0.2454 | 0.2097 |
| personalized | 0.7065 | 0.2395 | 0.0032 |
| weekend*personalized | -0.4957 | 0.3352 | 0.1392 |
| *Two-way interaction 3* | β | SE | p |
| Intercept | -0.9808 | 0.1748 | <.0001 |
| Am | 0.1452 | 0.2446 | 0.5527 |
| personalized | 0.4965 | 0.2388 | 0.0376 |
| am*personalized | -0.0817 | 0.3344 | 0.8070 |

β: log odds ratio, SE: standard error, am denotes 7am vs. 7pm notification, weekend denotes Sat vs. Wed notification, personalized denotes personalized vs. standard notification
