# Supplementary material for: Increasing Engagement in the Electronic Framingham Heart Study: Factorial Randomized Controlled Trial
Source: J Med Internet Res. 2023 Jan 20;25:e40784. doi: 10.2196/40784 (PMC9898831; doi:10.2196/40784)
Supplement: Multimedia Appendix 9 [file jmir_v25i1e40784_app9.docx]

# Multimedia Appendix 9: Table S3. Odds ratios from the two-way interaction analyses for the proportion of participants transmitting at least one BP measurement within 7 days of each weekly notification

| Comparison | Stratum | OR (95%CI) | Interaction p |
| --- | --- | --- | --- |
| Sat vs Wed | 7am | 0.92 (0.58-1.46) | 0.46 |
| Sat vs Wed | 7pm | 1.18 (0.75-1.86) |  |
| Personalized vs standard | Wed | 2.03 (1.27-3.24) | 0.14 |
| Personalized vs standard | Sat | 1.24 (0.78-1.96) |  |
| Personalized vs standard | 7am | 1.64 (1.03-2.62) | 0.81 |
| Personalized vs standard | 7pm | 1.51 (0.96-2.40) |  |
