# Supplementary material for: Increasing Engagement in the Electronic Framingham Heart Study: Factorial Randomized Controlled Trial
Source: J Med Internet Res. 2023 Jan 20;25:e40784. doi: 10.2196/40784 (PMC9898831; doi:10.2196/40784)
Supplement: Multimedia Appendix 10 [file jmir_v25i1e40784_app10.docx]

# Multimedia Appendix 10: Table S4. Longitudinal analysis of the weekly proportion of participants transmitting at least one BP measurement within 7 days of each weekly notification

| Effect | Num DF | Den DF | F Value | Pr > F |
| --- | --- | --- | --- | --- |
| *Personalized vs. standard notification* | | | | |
| personalized | 1 | 14679 | 18.01 | <.0001 |
| week | 1 | 14679 | 69.44 | <.0001 |
| personalized*week | 1 | 14679 | 4.10 | 0.0428 |
| *Morning vs. evening notification* | | | | |
| am | 1 | 14679 | 0.01 | 0.9412 |
| week | 1 | 14679 | 75.95 | <.0001 |
| am*week | 1 | 14679 | 4.77 | 0.0289 |
| *Weekend vs. weekday notification* | | | | |
| weekend | 1 | 14679 | 0.13 | 0.7182 |
| week | 1 | 14679 | 65.67 | <.0001 |
| weekend*week | 1 | 14679 | 1.16 | 0.2813 |

am denotes 7am vs. 7pm notification, weekend denotes Sat vs. Wed notification, personalized denotes personalized vs. standard notification
