# Supplementary material for: Increasing Engagement in the Electronic Framingham Heart Study: Factorial Randomized Controlled Trial
Source: J Med Internet Res. 2023 Jan 20;25:e40784. doi: 10.2196/40784 (PMC9898831; doi:10.2196/40784)

# Multimedia Appendix 11: Figure S3. Proportion of participants transmitting at least one BP measurement within 7 days of each weekly notification in the morning and evening notification groups


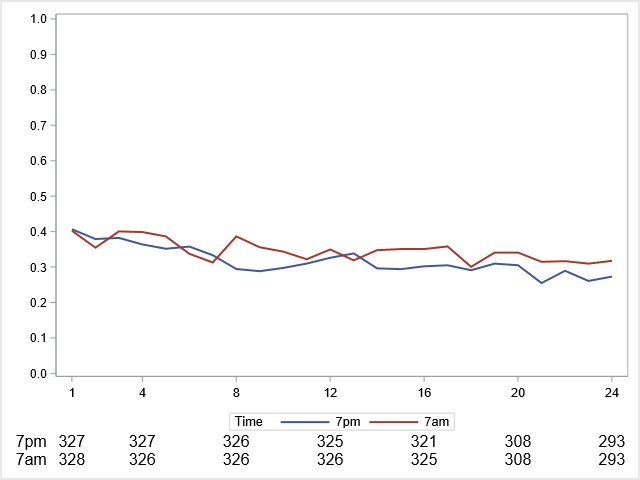

Supplement: Multimedia Appendix 11 [file jmir_v25i1e40784_app11.docx]
