# Supplementary material for: Increasing Engagement in the Electronic Framingham Heart Study: Factorial Randomized Controlled Trial
Source: J Med Internet Res. 2023 Jan 20;25:e40784. doi: 10.2196/40784 (PMC9898831; doi:10.2196/40784)
Supplement: Multimedia Appendix 13 [file jmir_v25i1e40784_app13.docx]

# Multimedia Appendix 13: Table S5. Three-way and two-way interaction analyses for the proportion of participants transmitting at least one HR measurement within 7 days of each weekly notification

|  | β | SE | p |
| --- | --- | --- | --- |
| *Three-way interaction* |  |  |  |
| Intercept | 0.4661 | 0.2255 | 0.0388 |
| weekend | 0.0840 | 0.3216 | 0.7940 |
| am | -0.0400 | 0.3202 | 0.9006 |
| weekend*am | 0.3549 | 0.4629 | 0.4432 |
| personalized | 0.1720 | 0.3247 | 0.5963 |
| weekend*personalized | -0.00985 | 0.4640 | 0.9831 |
| am*personalized | 0.2843 | 0.4648 | 0.5408 |
| weekend*am*personalized | -0.9666 | 0.6598 | 0.1429 |
| *Two-way interaction 1* | β | SE | p |
| intercept | 0.5500 | 0.1621 | 0.0007 |
| weekend | 0.0786 | 0.2317 | 0.7345 |
| am | 0.0974 | 0.2313 | 0.6735 |
| weekend*am | -0.1325 | 0.3281 | 0.6863 |
| *Two-way interaction 2* | β | SE | p |
| intercept | 0.4463 | 0.1601 | 0.0053 |
| weekend | 0.2561 | 0.2309 | 0.2674 |
| personalized | 0.3120 | 0.2321 | 0.1788 |
| weekend*personalized | -0.4936 | 0.3288 | 0.1333 |
| *Two-way interaction 3* | β | SE | p |
| Intercept | 0.5076 | 0.1607 | 0.0016 |
| Am | 0.1305 | 0.2305 | 0.5713 |
| personalized | 0.1669 | 0.2319 | 0.4719 |
| am*personalized | -0.2020 | 0.3283 | 0.5384 |

β: log odds ratio, SE: standard error; am denotes 7am vs. 7pm notification, weekend denotes Sat vs. Wed notification, personalized denotes personalized vs. standard notification
