# Supplementary material for: Increasing Engagement in the Electronic Framingham Heart Study: Factorial Randomized Controlled Trial
Source: J Med Internet Res. 2023 Jan 20;25:e40784. doi: 10.2196/40784 (PMC9898831; doi:10.2196/40784)
Supplement: Multimedia Appendix 14 [file jmir_v25i1e40784_app14.docx]

# Multimedia Appendix 14. Table S6. Odds ratios from the two-way interaction analyses for the proportion of participants transmitting at least one HR measurement within 7 days of each weekly notification

| Comparison | Stratum | OR (95%CI) | Interaction p |
| --- | --- | --- | --- |
| Sat vs Wed | 7am | 1.08 (0.69-1.70) | 0.69 |
| Sat vs Wed | 7pm | 0.95 (0.60-1.49) |  |
| Personalized vs standard | Wed | 1.37 (0.87-2.15) | 0.13 |
| Personalized vs standard | Sat | 0.83 (0.53-1.32) |  |
| Personalized vs standard | 7am | 1.18 (0.75-1.86) | 0.54 |
| Personalized vs standard | 7pm | 0.97 (0.61-1.52) |  |
