# Supplementary material for: Increasing Engagement in the Electronic Framingham Heart Study: Factorial Randomized Controlled Trial
Source: J Med Internet Res. 2023 Jan 20;25:e40784. doi: 10.2196/40784 (PMC9898831; doi:10.2196/40784)
Supplement: Multimedia Appendix 15 [file jmir_v25i1e40784_app15.docx]

# Multimedia Appendix 15: Table S7. Longitudinal analysis of the weekly proportion of participants transmitting at least one HR measurement within 7 days of each weekly notification

| Effect | Num DF | Den DF | F Value | Pr > F |
| --- | --- | --- | --- | --- |
| *Personalized vs. standard notification* | | | | |
| personalized | 1 | 14587 | 4.89 | 0.0270 |
| week | 1 | 14587 | 64.34 | <.0001 |
| personalized*week | 1 | 14587 | 5.40 | 0.0202 |
| *Morning vs. evening notification* | | | | |
| am | 1 | 14587 | 2.42 | 0.1201 |
| week | 1 | 14587 | 45.65 | <.0001 |
| am*week | 1 | 14587 | 0.37 | 0.5433 |
| *Weekend vs. weekday notification* | | | | |
| weekend | 1 | 14587 | 1.08 | 0.2982 |
| week | 1 | 14587 | 42.62 | <.0001 |
| weekend*week | 1 | 14587 | 0.02 | 0.8992 |

am denotes 7am vs. 7pm notification, weekend denotes Sat vs. Wed notification, personalized denotes personalized vs. standard notification
