# Supplementary material for: Increasing Engagement in the Electronic Framingham Heart Study: Factorial Randomized Controlled Trial
Source: J Med Internet Res. 2023 Jan 20;25:e40784. doi: 10.2196/40784 (PMC9898831; doi:10.2196/40784)
Supplement: Multimedia Appendix 18 [file jmir_v25i1e40784_app18.docx]

# Multimedia Appendix 18: Table S8. Survey completion among 205 individuals who had not been enrolled in eFHS prior to the beginning of the messaging trial.

|  | Personalized notification (n=102) | Standard notification (n=103) | P value |
| --- | --- | --- | --- |
| Baseline survey | | | |
| Complete | 94 (92) | 88 (85) | 0.25 |
| Partially complete | 2 (2) | 6 (6) |  |
| Missing | 6 (6) | 9 (9) |  |
| 3-month survey | | | |
| Complete | 60 (59) | 59 (57) | 0.83 |
| Partially complete | 0 | 0 |  |
| Missing | 42 (41) | 44 (43) |  |
